# Chemical Analysis and Transplacental Transfer of Oseltamivir and Oseltamivir Carboxylic Acid in Pregnant Rats

**DOI:** 10.1371/journal.pone.0046062

**Published:** 2012-10-03

**Authors:** Chia-Chun Lin, Jiin-Cherng Yen, Yu-Tse Wu, Lie-Chwen Lin, Tung-Hu Tsai

**Affiliations:** 1 Institute of Pharmacology, School of Medicine, National Yang-Ming University, Taipei, Taiwan; 2 Institute of Traditional Medicine, School of Medicine, National Yang-Ming University, Taipei, Taiwan; 3 National Research Institute of Chinese Medicine, Taipei, Taiwan; 4 Graduate Institute of Acupuncture Science, China Medical University, Taichung, Taiwan; 5 Department of Education and Research, Taipei City Hospital, Taipei, Taiwan; University of Cambridge, United Kingdom

## Abstract

In view of the limited information on the pharmacokinetics of oseltamivir (OSE) during pregnancy, this study aims to evaluate the placental transportation of OSE and its active metabolite oseltamivir carboxylic acid (OCA) in rats. A validated liquid chromatography-tandem mass spectrometry (LC-MS/MS) system coupled to an *in vivo* transplacental model has been developed to determine OSE and OCA in the placenta, amniotic fluids and fetus of 13-day pregnant Sprague-Dawley rats. Concentrations of OSE and OCA in plasma, amniotic fluids, placenta, and fetus were measured by the validated LC-MS/MS after OSE administration (10 mg/kg, iv). The pharmacokinetic data of both analytes were examined by non-compartmental modeling. The area under the concentration-time curve (AUC) of OCA in maternal plasma was found to be 3.6 times larger than that of OSE. The AUCs of OCA in both amniotic fluid and fetus were significantly decreased, in comparison with that in maternal plasma (reduced by 76.7 and 98.1%, respectively). We found that both OSE and OCA can penetrate the placenta, amniotic fluids and fetus in rats during pregnancy; however, the penetration of OCA was much lower than that of OSE. The mother-to-fetus transfer ratio was defined as AUC_fetus_/AUC_mother_. The data demonstrated that the mother-to-fetus transfer ratio of OSE and OCA were 1.64 and 0.019, respectively, suggesting that OSE, but not OCA, penetrated through the placenta. Moreover, OCA might not be easily metabolized in the fetus due to the lack of carboxylase in the fetus.

## Introduction

A new subtype of influenza A (H1N1) virus resulted in the outbreak of an influenza pandemic, referred to as pandemic H1N1, in 2009. Generally, influenza pandemics cause severe illness with high incidences of morbidity and mortality. High-risk populations include very young children, the elderly and pregnant women. Of these high-risk populations, pregnant women must be especially cautious because of the high risk of influenza-related complications in their unborn babies [1]. Hence, healthcare agencies recommended OSE as the choice of drug for the treatment of influenza during pregnancy. The OSE phosphate (Tamiflu®) is an antiviral drug and an ethyl ester prodrug of the active neuraminidase inhibitor, OCA. It is rapidly hydrolyzed by a liver enzyme, carboxylesterase, to become the active metabolite, OCA. Chemically, the antiviral mechanism of OSE is to block the release of virus from infected host cells of the sialic acid analogue structure [2]. OSE is usually administered orally for the treatment and prophylaxis of influenza virus infection [3].

According to preclinical studies and post-marketing surveillance, OSE and its active metabolite have no direct effects on genotoxicity, teratology, or carcinogenicity [4], [5]. There were also no adverse effects on the development of embryos or fetuses when rabbits and rats were treated with dosages of up to 1500 and 500 mg/kg/day, respectively [4]. However, some adverse events like abortion and malformation have been reported and can occur during pregnancy or in fetal development after pregnant patients received OSE therapy [5]. Although OSE is not thought not to possess adverse effects on pregnancy or fetal development, physicians should be aware of the available safety information and the health conditions of patients when they prescribe OSE for pregnant women [6]. A previous study has shown that the transplacental transfer of OCA was incomplete and accumulation was minimal, even at very high doses of OSE, though the finding as obtained in an *ex vivo* human transplacental model [7]. Recent report (2012) indicated that the transplacental transfer of OSE and OCA could be observed in a human opened and perfused placental cotyledon model while the perfused concentrations of OSE and OCA were similar to the maximal concentrations of OSE and OCA in clinical human plasma [8].

In September 2009, an eight-month pregnant woman was infected and tested positive for the new strain of the H1N1 virus during the outbreak of swine flu in Taitung County, Taiwan. She declined and insisted on not taking Tamiflu for fear of the possible effect of the medication on her baby. Her health deteriorated rapidly and she eventually died. Due to the insufficient information of oseltamivir, pregnant women during pandemic period would be concerned of the safety and effect of the antiviral agents on their fetus. In other words, these pregnant women care about the health of the fetus. Hence, the compliance of pregnant women for the use of antiviral medications is low [9]. To date, there has been no experimental pregnant animal model for investigating the penetration of OSE and OCA into amniotic fluid, placenta and fetus. To make up for such deficiency, the aim of study is to develop an experimental model for examining transplacental transfer of OSE and OCA.

Quantification methods for OSE and OCA detection include micellar electrokinetic chromatography [10] and HPLC with UV [7], [11], [12] or tandem mass spectrometric detection [13]–[15]. These methods are employed to analyze the concentrations of OSE and OCA in different kinds of biological fluids and tissues. Currently, liquid chromatographic systems combined with a tandem mass spectrometric method are the primary means of determining OSE or OCA in human and animal plasma, saliva, urine, cerebrospinal fluid and brain [13], [16]. However, there are no validated methods for measuring OSE and OCA in amniotic fluid, placenta and fetus. This study, therefore, develops an HPLC-MS/MS method for the analysis of OSE and OCA in plasma, amniotic fluid, placenta and fetus to investigate the transplacental transfer of OSE and OCA in animal.

## Materials and Methods

### Isolation and purification of OSE phosphate

Ethyl (3R,4R,5S)-4-acetylamino-5-amino-3(1-ethylpropoxy)1-cyclohexene-1- carboxylate phosphate, OSE phosphate, Tamiflu®) was isolated and purified from Tamiflu capsules (F. Hoffmann-La Roche, Basel, Switzerland). The isolation and purification process is briefly described below. A total 70 expired capsules of Tamiflu® were collected and crushed to powder. The powder was dissolved in 200 mL water and then basified using ammonium water to pH 10. The basified solution was extracted two times by ethyl acetate (200 mL). The ethyl acetate extract was then combined and concentrated to yield OSE. OSE (4.7 g) was then dissolved in ethanol and filtered by paper to remove undissolved residuals. The ethanol volume was reduced to 20 mL by heating. The OSE ethanol solution was added into phosphoric acid (1.7 g) under stirring and heated at 50°C for 30 minutes. After returning to room temperature, the solution was left standing in the refrigerator (4°C) for crystal precipitation. OSE phosphate crystals were separated from the solution, and their spectral data (NMR, MS, and UV) were determined, which agreed with the data in the literature [17].

The OCA was purchased from Toronto Research Chemicals Inc. (Ontario, Canada). Acetonitrile (Baker analyzed LC/MS reagent) and ammonium hydroxide (aq, 28.0–30.0%, NH_4_OH) were purchased from J.T. Baker (Phillipsburg, NJ, USA). Formic acid, methanol, ammonium acetate and sodium chloride of HPLC grade or GR for analysis grade were purchased from E. Merck (Darmstadt, Germany). Perchloric acid (70%, HClO_4_) was from Junsei Chemical Co. (Tokyo, Japan). Lidocaine hydrochloride injection solution (20 mg/mL), used as an internal standard, was from Taiwan Shiteh Organic Pharmaceutical Co. (Taipei, Taiwan). Heparin sodium was purchased from Sigma-Aldrich (St. Louis, MO, USA). Pure water for all preparations was obtained using the Milli-Q system (Millipore, Milford, MA, USA).

### Experimental animal

The protocol listed below has been reviewed and approved by the Institutional Animal Care and Use Committee (IACUC; approval number 980817) by the Institutional Animal Experimentation Committee of the National Yang-Ming University. Female Sprague-Dawley rats, who were pregnant for 13 days with weight ranging between 300 and 350 g (from the Laboratory Animals Center of National Yang-Ming University), were used in this study. Then, these rats adapt into the environment after transportation in around 3 days before experiment [18], [19]. The previous reports demonstrated that this gestation stage has been recommended on the placenta penetration study. The animals were housed in standard cages kept in a temperature-controlled room with a regular light and dark cycle. Free access to food (Laboratory Rodent Diet 5001, PMI Feeds, Richmond, IN) and water was available at all times.

Pregnant rats were anesthetized with a mixed solution of 1 g/mL urethane and 0.1 g/mL α-chloralose (1 mL/kg, intraperitoneal) and remained anesthetized as needed throughout the experimental period. The maternal rat was placed on its back with the lower half of its body immersed in a warm bath of isotonic saline at 37°C in order to maintain the body temperature. After laparotomy, the laparotomy incision was covered using gauze immersed with warm saline. To investigate the transplacental transfer of OSE and OCA, OSE solution (10 mg/mL) prepared in a 0.9% sodium chloride solution was administered via the femoral vein by bolus injection at a dose of 10 mg/kg. Biological samples of blood, amniotic fluid, placenta and fetus were collected at different time points at 5, 15, 30, 60, 120, 180, 240, and 360 min after drug administration. At each time point, we collected amniotic fluid, one placenta and one fetus from one single uterus of a dam until the end of experiment. Collected placenta and fetus samples were weighed immediately, and all samples were stored at −20°C for further sample preparation.

### Preparation of plasma, amniotic fluid, placenta and fetus samples

The solid phase extraction (SPE) procedure, which was modified from a previous report [20], was employed to extract the analytes from plasma and amniotic fluid samples. In brief, an aliquot of 100 μL plasma with 10 µL of internal standard solution (lidocaine, 25 ng/mL) was mixed well with 400 µL of 0.5 M HClO_4_, and the mixture was centrifuged at 10,000×g. The supernatant was introduced into a SPE cartridge (Strata-X-C, 1 mL, 30 mg) that had already been conditioned and acidified with 1 mL of methanol and 1 mL of 2% (v/v) formic acid. Cartridges were washed twice with 1 mL of 2% (v/v) formic acid in water, followed by 1 mL of methanol. Finally, the analytes were eluted three times with 1 mL methanol with 5% NH_4_OH. The eluent was dried at 35°C and then reconstituted with 100 µL of the mixture of 80% aqueous acetonitrile (80% of acetonitrile, v/v) before analysis.

One part (g) of the chopped placenta or fetus was homogenized with a two-fold amount of 0.9% sodium chloride solution, using a Polytron PT 2100 homogenizer (Kinematica, Lucerne, Switzerland). The suspension was further sonicated for 10 min and then centrifuged at 10,000×g for 5 min. The supernatant (100 µL) was added with internal standard solution (10 µL), 0.5 M HClO_4_ (400 µL) and then extracted by the SPE processes. Finally, the reconstituted sample was analyzed by HPLC-MS/MS.

### High-performance liquid chromatography and method validation

The ACQUITY UPLC system consisted of a quaternary pump, an autosampler and a column oven (Waters, MA, USA). The analytes were separated by a ZIC-HILIC column (150×2.1 mm, 3.5 μm, Merck) protected by a ZIC-HILIC guard column (20×2.1 mm, 5 μm, Merck) with an isocratic elution using a mobile phase comprising 10 mM ammonium acetate (pH 6.0) and acetonitrile (17∶83, v/v). The flow rate was 0.3 mL/min and the injection volume was 2 μL. The mass spectrometer was a triple quadrupole Xevo TQ MS from Waters (MA, USA). An electrospray ionization (ESI) source in the positive-ionization mode was used in the experiments. Mass spectrometer conditions were optimized and set as follows: cone voltage 10 V and collision energy 20 eV for OSE; and cone voltage 40 V and collision energy 10 eV for OCA; cone voltage 20 V and collision energy 20 eV for internal standard. The capillary voltage was set at 0.6 kV. Source and desolvation temperatures were set at 150°C and 500°C, respectively. The desolvation and the cone gas were 1000 L/h and 20 L/h, respectively. Multiple reaction monitoring (MRM) and the transition of precursor to product ion were used and monitored at *m*/*z* 313.2→165.9 for OSE, *m*/*z* 285.2→197.1 for OCA, *m*/*z* 235.1→86.0 for internal standard. The software MassLynx V4.1 (Waters) was used for data processing.

Validation of the analytical methods described in this study included linearity, accuracy, precision, matrix effect and recovery. All validation tests were performed on these two analytes in the different matrices. Standard solutions of OSE and OCA were prepared in methanol to make 1 mg/mL as stock solution, which was further diluted to give a series of working standard solutions. Lidocaine solution (25 ng/mL) was used as internal standard. Calibration curves were prepared by adding these working standard solutions into blank plasma, amniotic fluid or tissue homogenates to give calibration concentrations of 1, 10, 50, 100, and 500 ng/mL. The subsequent SPE clean-up procedures have been described above.

Linearity was evaluated by coefficient of determination of (*r* ^2^) of with at least 0.995 to be considered acceptable. Precision and accuracy within the calibration range was estimated at three concentrations of 25, 250 and 500 ng/mL in three runs daily for three consecutive days. Calibration curves were calculated by the nominal concentration of OSE and OCA calibration standards versus the peak area ratio of OSE and OCA to internal standard. Sensitivity of analytes was determined by calculating the signal-to-noise ratio of the lowest limit of quantitation (LLOQ) of samples. The limits of detection (LOD) and quantification (LOQ) were determined at S/N (the ratio of signal to noise) of 3 and 10 under the present chromatographic conditions. The limit of quantification (LOQ) for OSE and OCA was 1 ng/mL, according to the deviation of response for the linear range (≤20%). The concentration of any sample higher than the calibration range was corrected by dilution with blank matrix containing internal standard. Accuracy and precision were evaluated by the nominal concentration (C_nom_) or the observed concentration (C_obs_). Accuracy was expressed as: accuracy (bias, %)  =  [(C_obs_ – C_nom_)/C_nom_] ×100, while precision was expressed as: precision (RSD, %)  =  [standard deviation (SD)/C_obs_] ×100.

The matrix effect and recovery for the quantification of OSE and OCA were evaluated by three sets of samples [21]. For Set 1, stock solutions of OSE, OCA and internal standard were prepared by 80% aqueous acetonitrile. These samples were then transferred into sample vials, and 2 μL of these samples were injected directly into the HPLC-MS/MS system. Set 2 comprised post-extraction fortified samples prepared by adding 10 μL standard solutions of the two analytes into 90 μL reconstituted extract solutions from three different lots of each blank biological matrix to obtain the concentrations of 2.5, 25 and 250 ng/mL of OSE and of OSE. After through mixing, samples of this set were transferred into sample vials, and 2 μL was injected into the HPLC-MS/MS system. Set 3 included pre-extraction fortified samples used for calibration. The samples were prepared by the same procedures as those for Set 2. The OSE and OCA were spiked with the blank biological matrices to obtain the concentrations of 2.5, 25 and 250 ng/mL. After SPE extraction and reconstitution, these samples were loaded into sample vials, and 2 μL of the sample solution was injected into the HPLC-MS/MS system.

Recovery was evaluated by the peak area ratio of analytes to internal standard for extracted QC samples (Set 3) compared with the peak area ratio of analytes to internal standard, which was spiked after 0.5 M HClO_4_ treatment, followed by SPE extraction (Set 2) and multiplied by 100. Matrix effects were determined by comparing the peak area ratio of analytes to internal standard spiked post-SPE extraction (Set 2) with the peak area ratio of analytes to internal standard (Set 1) and multiplied by 100.

### Pharmacokinetic analysis and statistics

Curves of various biological concentration data versus sampling time for OSE and OCA were constructed by their corrected pharmacokinetic curves and then processed by the WinNonlin Standard Edition Version 1.1 (Scientific Consulting Inc., Apex, NC). The pharmacokinetic parameters for plasma sample were calculated by a noncompartmental model. In addition, an extravascular input noncompartmental model was employed to obtain pharmacokinetic parameters for amniotic fluid, placenta, and fetus. All data are presented as mean ± standard deviation (SD). Student's *t* test was used to evaluate differences and a value of P<0.05 was taken as statistically significant.

## Results and Discussion

### Method validation

**Figure 1:**
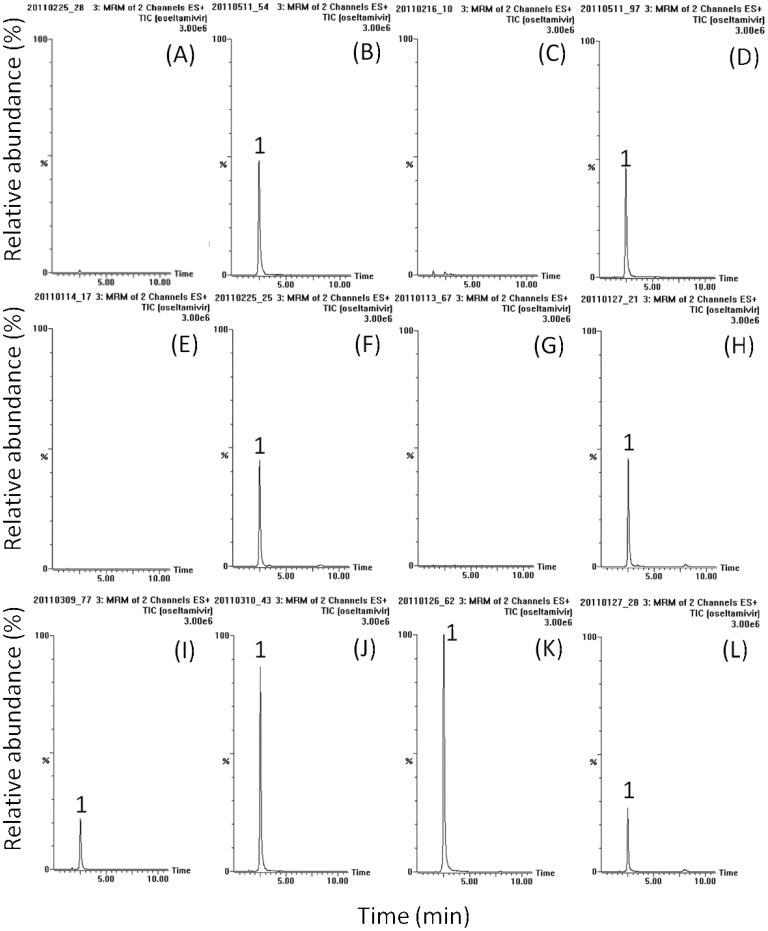
Typical LC-MS/MS chromatograms of oseltamivir in various biological samples of rat (A) blank plasma, (B) blank plasma containing oseltamivir (50 ng/mL), (C) blank amniotic fluid, (D) blank amniotic fluid containing oseltamivir (50 ng/mL), (E) blank placenta homogenate, (F) blank placenta homogenate containing oseltamivir (50 ng/mL), (G) blank fetus homogenate, (H) blank fetus homogenate containing oseltamivir (50 ng/mL), (I) maternal plasma sample with 20-times dilution after oseltamivir administration (10 mg/kg, iv), (J) amniotic fluid sample with 4-times dilution after oseltamivir administration (10 mg/kg, iv), (K) placenta homogenate sample after oseltamivir administration (10 mg/kg, iv), (L) fetus homogenate sample after oseltamivir administration (10 mg/kg, iv). 1: oseltamivir.

**Figure 2:**
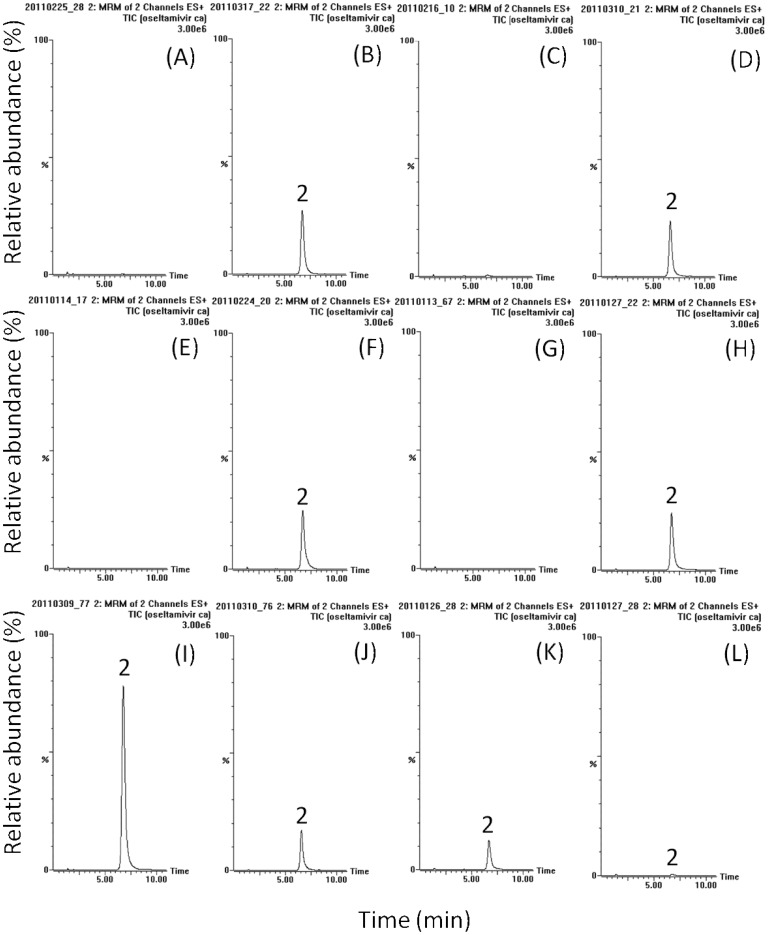
Typical LC-MS/MS chromatograms of oseltamivir carboxylic acid in various biological samples of rat (A) blank plasma, (B) blank plasma containing oseltamivir carboxylic acid (50 ng/mL), (C) blank amniotic fluid, (D) blank amniotic fluid containing oseltamivir carboxylic acid (50 ng/mL), (E) blank placenta homogenate, (F) blank placenta homogenate containing oseltamivir carboxylic acid (50 ng/mL), (G) blank fetus homogenate, (H) blank fetus homogenate containing oseltamivir carboxylic acid (50 ng/mL), (I) maternal plasma sample with 20-times dilution after oseltamivir administration (10 mg/kg, iv), (J) rat amniotic fluid sample with 4-times dilution after oseltamivir administration (10 mg/kg, iv), (K) placenta homogenate sample after oseltamivir (10 mg/kg, iv), (L) fetus homogenate sample at 5 min after oseltamivir administration (10 mg/kg, iv). 2: oseltamivir carboxylic acid.

**Figure 3:**
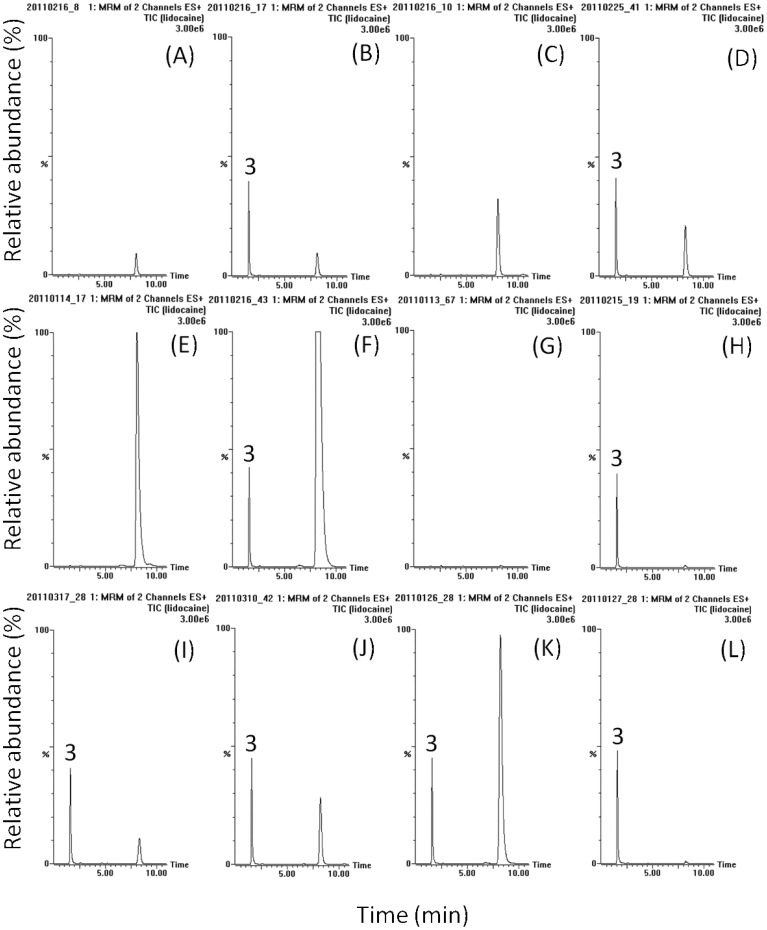
Typical LC-MS/MS chromatograms of lidocaine (internal standard) in various biological samples of rat (A) blank plasma, (B) blank plasma containing lidocaine (2.5 ng/mL), (C) blank amniotic fluid, (D) blank amniotic fluid containing lidocaine (2.5 ng/mL), (E) blank placenta homogenate, (F) blank placenta homogenate containing lidocaine (2.5 ng/mL), (G) blank fetus homogenate, (H) blank fetus homogenate containing lidocaine (2.5 ng/mL), (I) maternal plasma sample with 20-times dilution after oseltamivir administration (10 mg/kg, iv), (J) amniotic fluid sample with 4-times dilution after oseltamivir administration (10 mg/kg, iv), (K) placenta homogenate sample at 5 min after oseltamivir administration (10 mg/kg, iv), (L) fetus homogenate sample at 5 min after oseltamivir administration (10 mg/kg, iv). 3: lidocaine.

OSE, OCA and the internal standard were detected in the positive MRM mode and their retention times were approximately 2.5, 6.6 and 1.6 min, respectively. The mass spectra revealed peaks of OSE and OCA at m/z 313.2 and 285.2 corresponding to [M+H]^+^. The product ions m/z 165.9 and 197.1 of these two analytes were selected for quantification. Figures 1, 2 and 3 show the typical HPLC–MS/MS chromatograms for plasma, amniotic fluid, placenta, and fetus with their respective blank matrix, blank matrix spiked with OSE, OCA and internal standard, and samples collected after OSE administration. Chromatograms of the blank biological samples show no significant interference in the analyte-free samples.

The analytical method was validated by precision and accuracy within both interday and intraday assays. Additionally, the assays of recovery and matrix effect were used both to assess the influence of the biological matrix on the mass signals of analytes and to estimate the amount of each analyte collected after sample preparation. The calibration curves were linear over the concentration range of 1–500 ng/mL for OSE and OCA in all biological samples. Linearity was assessed by the coefficients of determination (*r* ^2^) obtained for the regression line. The limit of quantification (LOQ) for OSE and OCA was 1 ng/mL, according to the deviation of response for the linear range (≤20%). Tables 1 and 2 show the accuracy and precision of these analytes during intraday and interday assays, respectively. For OSE analysis, the precision defined by RSD ranged from 0.9 to 11.2%; while the accuracy, expressed as the percentage of difference between the mean observed values and the nominal concentration, was in the range of −8.5 and 9.0%. For OCA, the precision of analysis method ranged from 1.7 to 11.7% while the bias for accuracy was within the range of −10.8 to 14.6%. These results show that the precision and accuracy of this analytical method were less than 20%, indicating that the method was reproducible.

**Table 1:** Accuracy and precision values of the HPLC-MS/MS method for the determination of oseltamivir in rat plasma, amniotic fluid and tissue homogenate.

Nominal concentration (ng/mL)	Intraday-assay		Interday-assay	
	Precision (%)	Accuracy (%)	Precision (%)	Accuracy (%)
Plasma				
25	9.9	6.8	5.7	−3.6
250	3.8	8.6	8.9	1.8
500	4.9	5.8	9.1	2.9
Amniotic fluid				
25	1.4	3.3	2.0	2.4
250	9.9	3.1	11.2	4.4
500	1.4	1.8	6.6	3.0
Placenta				
25	0.9	8.4	1.7	7.1
250	1.6	1.0	4.7	−8.5
500	4.2	0.3	6.2	−1.6
Fetus				
25	4.4	−0.1	3.8	−1.8
250	1.3	7.7	5.8	2.1
500	3.6	7.8	3.6	9.0

**Table 2:** Accuracy and precision values of the HPLC-MS/MS method for the determination of oseltamivir carboxylic acid in rat plasma, amniotic fluid and tissue homogenate.

Nominal concentration (ng/mL)	Intraday-assay		Interday-assay	
	Precision (%)	Accuracy (%)	Precision (%)	Accuracy (%)
Plasma				
25	3.4	3.2	3.3	−3.6
250	2.2	12.5	8.0	4.0
500	2.6	−5.8	2.6	−10.8
Amniotic fluid				
25	4.7	2.4	11.7	−2.6
250	3.9	0.8	4.1	2.0
500	1.7	−0.6	4.7	−7.1
Placenta				
25	7.3	5.1	8.2	1.1
250	2.0	9.6	4.6	5.6
500	5.3	−0.7	5.2	−2.3
Fetus				
25	4.0	14.6	3.6	−1.8
250	6.0	−1.0	5.2	8.5
500	6.1	1.5	6.1	−0.6

To examine the matrix effect of the analytes in various biological samples, the post-extraction fortification method was employed to examine the matrix effect of the analytes in different biological samples. Results obtained using the post-extraction fortification method show that the average matrix effects of OSE in plasma, amniotic fluid, placenta, and fetus were 96.5±0.5, 98.9±2.3, 88.5±7.2, and 89.2±2.0%, respectively (Table 3), while the average matrix effects of OCA in plasma, amniotic fluid, placenta, and fetus were 91.9±4.6, 99.0±2.2, 98.6±6.5, and 101.2±1.5%, respectively (Table 4). The present study is the first research on the determination of OSE and OCA in rat amniotic fluid, placenta, and fetus. The above results indicate that the placenta samples had the most severe ion suppression in the analysis of OSE. Hence, in order to maintain the same matrix effect as the original samples and to measure correctly the concentrations of analytes, the samples exceeding the calibration range were diluted by the extracted blank matrices containing internal standard. Moreover, the recovery data for the analytes in various biological samples are shown in Table 3. The average recoveries for OSE were 91.3±19.4% for plasma, 90.3±13.5% for amniotic fluid, 104.7±14.5% for placenta, and 97.7±5.8% for fetus. In addition, the recoveries for OCA were higher than 90% in those biological samples (Table 4).

**Table 3:** Matrix effect (ME) and recovery (RE) data for oseltamivir in rat plasma, amniotic fluid and tissue homogenates.

Nominal concentration (ng/mL)		Set 1 a	Set 2 a	Set 3 a	ME (%)^b^	RE (%)^c^
Plasma	2.5	0.18±0.05	0.17±0.08	0.12±0.01	6.5	2.0
	25	1.76±0.02	1.69±0.05	1.54±0.05	5.6	1.2
	250	18.5±0.62	18.0±1.96	19.9±0.28	7.4	11
	Average				96.5±0.9	91.3±19.4
Amniotic fluid	2.5	0.18±0.05	0.18±0.01	0.14±0.03	01	7.5
	25	1.76±0.02	1.79±0.09	1.59±0.02	02	8.9
	250	18.5±0.62	17.4±3.29	18.2±2.22	4.2	05
	Average				98.9±4.0	90.3±13.5
Placenta	2.5	0.18±0.03	0.17±0.04	0.19±0.01	4.5	11
	25	1.90±0.01	1.40±0.36	1.61±0.01	3.5	15
	250	16.4±1.28	16.0±0.78	14.1±3.61	7.5	8.2
	Average				88.5±13.0	105±14.5
Fetus	2.5	0.18±0.03	0.16±0.02	0.16±0.02	9.4	9.9
	25	1.90±0.01	1.63±0.62	1.49±0.26	5.6	1.2
	250	16.4±1.28	15.2±2.80	15.5±0.29	2.5	02.1
	Average				89.2±3.4	97.7±5.8

a All values of Set 1, 2, and 3 were expressed as mean peak area ratio of analyte to internal standard (mean ± standard deviation) (n = 3).

b ME (%)  =  (mean peak area ratio of Set 2/mean peak area ratio of Set 1)×100.

c RE (%)  =  (mean peak area ratio of Set 3/mean peak area ratio of Set 2)×100.

**Table 4:** Matrix effect (ME) and recovery (RE) data for oseltamivir carboxylic acid in rat plasma, amniotic fluid and tissue homogenates.

Nominal concentration (ng/mL)		Set1 a	Set2 a	Set3 a	ME (%)^b^	RE (%)^c^
Plasma	2.5	0.18±0.02	0.19±0.07	0.15±0.01	01	3.1
	25	1.79±0.08	1.54±0.16	1.62±0.08	6.4	05
	250	17.0±0.74	15.0±4.04	13.8±4.11	8.2	1.8
	Average				91.9±7.8	93.3±10.9
Amniotic fluid	2.5	0.18±0.02	0.19±0.01	0.18±0.02	02	7.7
	25	1.79±0.08	1.69±0.08	1.54±0.05	4.6	0.9
	250	17.0±0.74	17.1±0.51	16.7±0.63	01	7.5
	Average				99.0±3.8	95.4±3.9
Placenta	2.5	0.166±0.00	0.17±0.06	0.16±0.07	9.6	3.7
	25	1.62±0.26	1.76±0.72	1.63±0.42	09	2.1
	250	16.3±4.40	14.2±4.39	13.5±0.30	6.9	5.0
	Average				98.6±11.1	93.6±1.5
Fetus	2.5	0.166±0.00	0.16±0.01	0.16±0.02	8.9	6.7
	25	1.62±0.26	1.68±0.23	1.63±0.28	04	7.1
	250	16.3±4.40	16.4±4.58	15.3±4.49	00	3.5
	Average				101±2.6	95.8±2.0

a All values of Set 1, 2, and 3 were expressed as mean peak area ratio of analyte to internal standard (mean ± standard deviation) (n = 3).

b ME (%)  =  (mean peak area ratio of Set 2/mean peak area ratio of Set 1)×100.

c RE (%)  =  (mean peak area ratio of Set 3/mean peak area ratio of Set 2)×100.

In order to validate the methods used in the analytical system, an appropriate internal standard is required. In previous analyses of OSE and OCA, trideuterated internal standards have usually been used to compensate for the matrix effect [13], [15]. Although using a trideuterated analyte as an internal standard is a means for assessing the matrix effect, to obtain and operate a deuterated internal standard requires special handling [22]. Because lidocaine has different LC behaviors and mass between OSE and OCA, the mass spectrometry can identify the analytes and the internal standard. Nevertheless, the present study used lidocaine as the internal standard and obtained acceptable results for matrix effect and recovery between these analytes. Previous reports have shown that good recoveries of OSE and OCA in rat plasma, urine, brain and cerebrospinal fluid can be obtained using on-line [16] or off-line [14], [15] SPE procedures. In this study, off-line SPE was used for sample preparation. Compared with previous methods, the proposed approach obtained good recoveries of OSE and OCA in different animal matrices (Tables 3 and 4). The average recovery was also greater than 90%, which is higher than that in previous reports [23].

### Pharmacokinetics of OSE and OCA in rats after OSE administration

**Figure 4:**
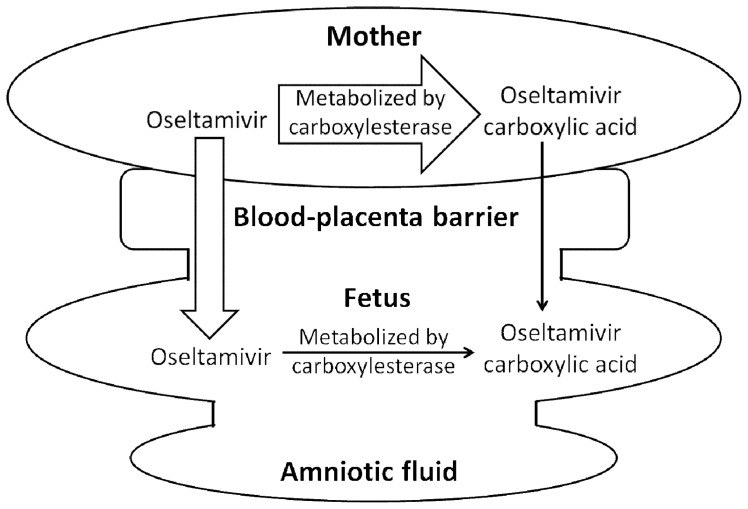
Scheme of *in vivo* animal model for transplacental transfer. Oseltamivir is rapidly biotransfered by carboxylesterase in the maternal tissues. Through blood-placenta barrier, oseltamivir and oseltamivir carboxylic acid can penetrate into fetus. The oseltamivir carboxylic acid in fetus may penetrate from the blood-placenta barrier or the metabolism of oseltamivir by carboxylase in the fetus. Oseltamivir and oseltamivir carboxylic acid can be also distributed over the amniotic fluid.

An *ex vivo* human placenta perfusion study has demonstrated that OSE can be detected and OCA is minimally accumulated in the fetus [7]. Worley *et* *al*. (2008) suggested that additional experiments are needed to characterize the pharmacokinetic behavior of OSE in pregnancy. Recently, a human placenta perfusion study by Berveiller *et* *al* (2012) showed that the fetal transfer rate is 8.5% and 6.6% for OSE and OCA, respectively [8]. There are some differences between these ex vivo models and the in vivo model. First, the experimental convenience is one of the major differences. Worley *et* *al*. (2008) and Berveiller *et* *al* (2012) investigated the transplacental transfer of OSE using the *ex vivo* perfused model of human placenta. Both of these two studies should consider the time of perfusion, and thus, they subsequently perfused the human placenta through vaginal delivery. Second, the differences in the perfused solution are also noteworthy. Worley *et* *al.* used the perfusate with a high concentration of albumin (30 g/L) and Berveiller *et* *al* used the perfusate with a low concentration of albumin (2 g/L). Here, we provide an *in vivo* experimental model to demonstrate transplacental transfer of OSE and OCA in pregnant rats. This model corresponded to the biological condition such as enzymes and proteins which maintain the regular functions in rat. In the *in vivo* animal model, every female rat has a Y-shaped uterus, which is divided into two parts, the sinister and dexter uterus. At designated time points, we collected the amniotic fluid, placenta, and fetus from a single uterus. Our data provided the first report for the penetration of OSE and OCA through the placenta into the amniotic fluid and fetus in a pregnant experimental animal (Figure 4).

**Figure 5:**
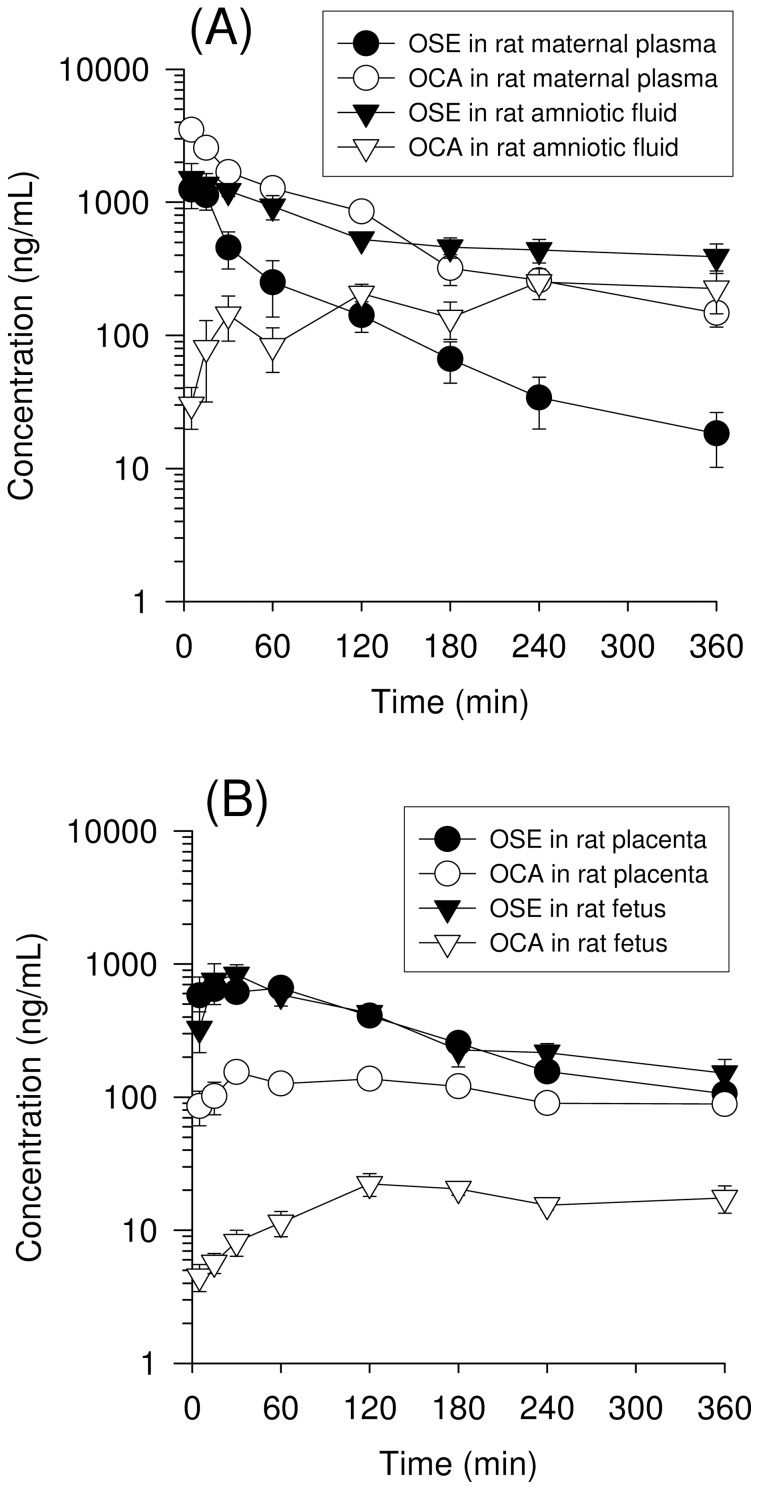
Concentration-time curves of oseltamivir (OSE) and oseltamivir carboxylic acid (OCA) after oseltamivir administration (10 mg/kg, iv) in various biological samples of rat (A) maternal plasma and amniotic fluid, (B) placenta and fetus. (n = 6).

The validated method was employed to determine the plasma and tissue distribution of OSE and OCA after OSE administration. The OSE dose (10 mg/kg) used in this study was about 1.5 times higher than the single clinical dosage of 75 mg orally [24]. The profiles of concentration vs. time for OSE and OCA in maternal plasma, amniotic fluid, placenta, and fetus are shown in Figure 5.

The areas under the concentration-time curves (AUC_0-t_) of OSE in maternal plasma and fetus were 1.27±0.68 hr μg/mL and 2.08±0.62 hr μg/mL, respectively (Table 5). The ratio of AUC_fetus_/AUC_plasma_, defined as mother-to-fetus transfer, was 1.64, suggesting that OSE was penetrated through the placenta into the fetus. On the other hand, the AUCs of OCA in maternal plasma and fetus were 4.62±0.70 hr μg/mL and 0.09±0.03 hr μg/mL, respectively (Table 5). The ratio of AUC_fetus_/AUC_plasma_ was 0.019, indicating that OCA resisted to penetrate through the placenta into the fetus. Another explanation for the high level of OSE in the fetus is the absence of the specific enzyme for converting OSE into OCA.

**Table 5:** Pharmacokinetic parameters of oseltamivir and oseltamivir carboxylic acid after oseltamivir administration (10 mg/kg, iv).

Parameter (units)	Maternal plasma	Amniotic fluid	Placenta	Fetus
*oseltamivir*				
t_1/2_ (hr)	1.71±1.09	3.16±1.37	1.92±0.54	1.98±0.69
AUC_0t_ (hr μg/mL)	1.27±0.68	3.56±0.82*	1.88±0.13	2.08±0.62
Vd (L/kg)	27.0±31.6	8.0±1.9	12.4±3.1	11.3±1.8
Cl (L/min/kg)	0.16±0.11	0.04±0.02*	0.08±0.01	0.08±0.04
*oseltamivir carboxylic acid*				
t_1/2_ (hr)	2.24±0.38	–	8.03±3.09*	12.0±10.1
AUC_0t_ (hr μg/mL)	4.62±0.70	1.08±0.15*	0.68±0.17*	0.09±0.03*
Vd (L/kg)	5.7±4.6	–	58.5±9.3*	407±133*
Cl (L/min/kg)	0.03±0.01	–	0.13±0.11	0.80±0.70

Area under the concentration versus time curve (AUC_0t_) is indicated. Values represent the means ± tandard deviation (n = 6).* P<0.05, compared with the same parameters in rat maternal plasma.

The concentration of OCA in maternal plasma was higher than that of OSE (Figure 5A). The AUC of OSE and OCA in maternal plasma was 1.27±0.68 hr μg/mL and 4.62±0.70 hr μg/mL, respectively (Table 5), which was about 3.6 times of OCA higher than OSE. These data suggested that OSE was easily hydrolyzed into OCA in the maternal site. The concentration-time curves and pharmacokinetic parameters for OSE and OCA in rat placenta and fetus are shown in Figure 5B, Table 5. These results described above demonstrate that OSE and OCA can pass through the blood-placental barrier and determined in the placenta, amniotic fluid, and fetus after OSE administration.

We found that the elimination half-time (t_1/2_) of OSE and OCA in maternal plasma of pregnant rat were 1.71±1.09 hr and 2.24±0.38 hr, respectively after OSE administration (Table 5). These results are consisted with previous findings that the elimination half-life of OCA is longer than that of OSE in rat [14], [25].

A rapid conversion from OSE to OCA was observed in the concentration-time profile (Figure 5A). A similar phenomenon was also observed in an earlier study [25], which can be attributed to rapid degradation by the enzyme of esterases [26]. The concentrations of OCA in amniotic fluid and fetus were much lower than in maternal plasma. The plasma protein binding rate for OSE and OCA is 42% and 3%, respectively [3]. Due to the relative weak binding rate of OSE and OCA, the transplacental transfer of unbound OSE and unbound OCA could be more constant and would not be greatly affected by plasma protein. Accordingly, the fetal transfer of OCA might be restricted by the blood-placental barrier, and the presence of OCA in fetuses might be attributed to hydrolysis of OSE by placental esterases [27], [28]. OSE is the substrate of P-glycoprotein (P-gp) [11] and P-gp transporters play an important role in transplacental transfer. During pregnancy, the expression of P-gp would increase with gestation progress [29]. However, there is no evidence proving that drug transporters are involved in the fetal transfer of OCA, though it has been reported that the transport of OCA via ATP-binding cassette subfamily B member 1 (ABCB1) should be minor [11]. Although OCA is a substrate of organic anion transporter 3 and multidrug resistance-associated protein (MRP) 4 [30], expression of these drug transporters was not found in the placenta [31]. Further investigation is needed to determine whether OCA is a substrate of multidrug resistance-associated proteins (MRP 1, 2 and 5), breast cancer resistance protein, and organic anion-transporting polypeptide 4 transporters located at the placental barrier.

Results of a previous enzymatic assay show that replications of the laboratory strains and of clinical isolates for influenza A and B viruses were inhibited by OCA, with the 50% inhibitory concentrations (IC_50_) of OCA being in the range of 0.3 to 2 nM (0.09 to 0.57 ng/mL) [32]. In the present study (Figure 5), the concentrations of OCA in maternal and fetal rat after injection of OSE were greater than IC_50_. Hence, the data demonstrated sufficient inhibition of influenza viruses in the fetus when the injection dose was 10 mg/kg.

OSE is an ester prodrug which will be catalyzed by carboxylesterase, human carboxylesterase (HCE) to the antiviral active metabolite OCA. A cell viability test demonstrated by Shi et al. (2006) suggested that OCA is more toxic than OSE [33]. In the case of pregnant woman concern in 2009, the OSE could hurt her baby. Our data demonstrate that only 1.9% of the more toxic metabolite OCA penetrated through the blood-placenta barrier into the fetus.

In conclusion, a sensitive, accurate and precise HPLC-MS/MS analytical method for the simultaneous quantification of OSE and OCA has been demonstrated. This assay system can be used for pharmacokinetics of OSE and OCA in rat plasma, amniotic fluid, placenta, and fetus. It presents no endogenous interference which would hinder the separation of analytes and it is sufficiently sensitive for the determination of analytes in biological samples. The animal experimental results demonstrate that both OSE and OCA can penetrate the placenta, amniotic fluid and fetus in a pregnant rat, though the mother-to-fetus transfer of OSE was much higher than that of OCA. The results show that rapid conversion to OCA occurred in maternal plasma after OSE administration and that the presence of OCA was observed immediately in maternal plasma. However, the OCA level in the fetus is about 1/50 of that presented in material plasma. Such difference is attributed to the absence of OSE metabolic enzyme in the fetus. These pharmacokinetic results provide a constructive contribution to better understand the placental transfer of OSE and OCA to support clinical application.
